# First person – Pearl Ryder

**DOI:** 10.1242/bio.057026

**Published:** 2020-10-26

## Abstract

First Person is a series of interviews with the first authors of a selection of papers published in Biology Open, helping early-career researchers promote themselves alongside their papers. Pearl Ryder is first author on ‘Quantitative analysis of subcellular distributions with an open-source, object-based tool’, published in BiO. Pearl conducted the research described in this article while a NIH-NRSA postdoctoral fellow in Dorothy Lerit's lab at Emory University School of Medicine, Atlanta, USA. She is now a postdoctoral fellow in Bioimage Analysis in the lab of Anne Carpenter at The Broad Institute, Cambridge, USA, investigating Extracting data and insights from biological images.


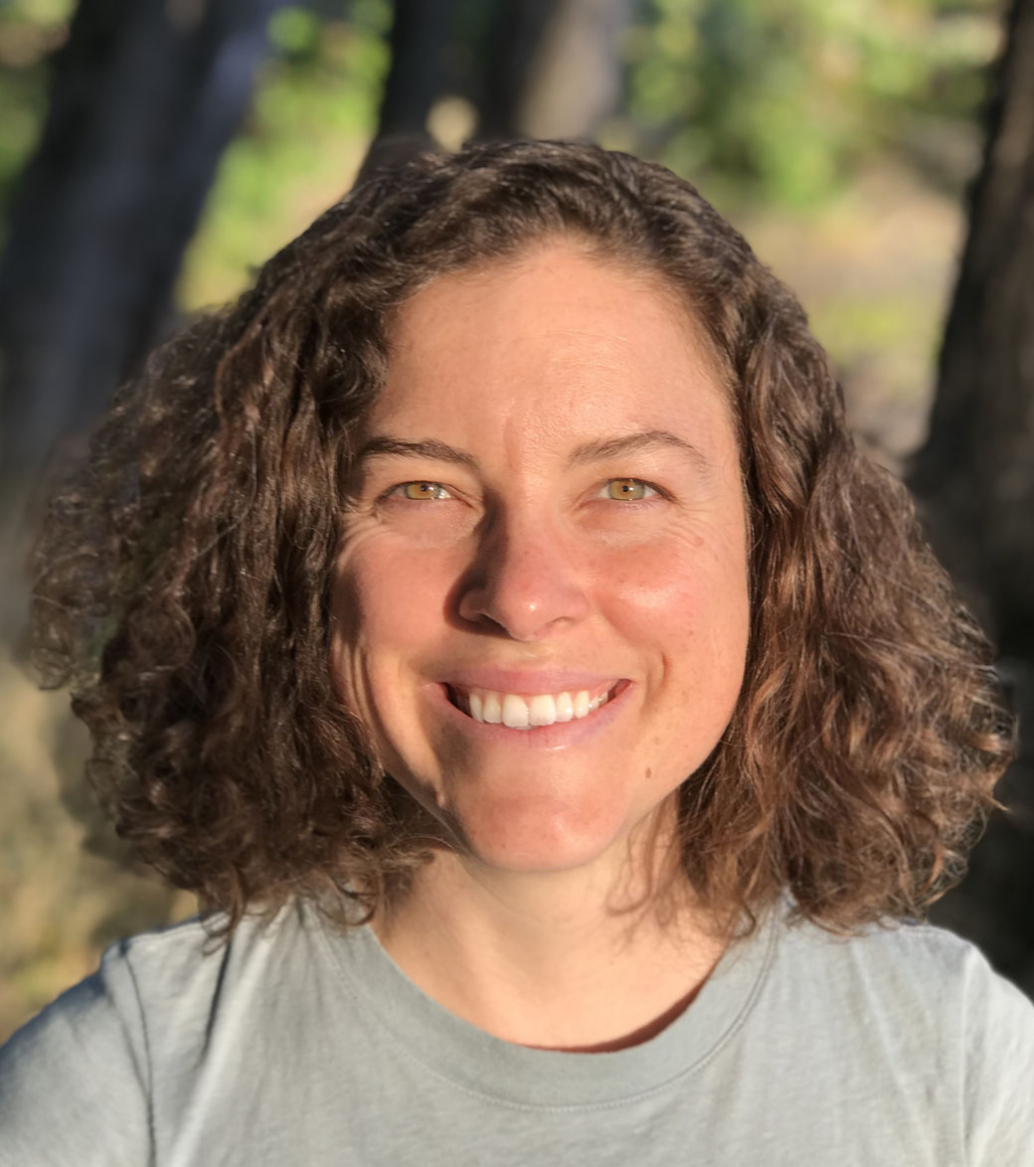


**Pearl Ryder, MD, PhD**

**What is your scientific background and the general focus of your lab?**

My interests are quite diverse, leading to a diverse scientific background. I am a cell biologist and a physician who is now training in computational image analysis. I love thinking about how proteins, sugars, lipids, and nuclei acids self-organize into functional units. During my postdoctoral fellowship in Dorothy Lerit's laboratory, I focused on an intriguing question: how and why does mRNA localize to centrosomes? Centrosomes are key regulators of cell shape and function (especially cell division), which is why we were interested in learning more about the regulation of this organelle. Tackling the question of mRNA localization to centrosomes led me to develop a software pipeline written in Python to quantitatively analyze my images of *Drosophila* embryos to test if mRNA was truly enriched at this subcellular structure. We realized that this tool could be useful to others and therefore decided to share it as an open source software tool and to describe it in this manuscript.

**How would you explain the main findings of your paper to non-scientific family and friends?**

Location, location, location. It's critical for real estate and for biological systems. For example, if a protein works on a target that only is present at one particular cell structure (such as at the nucleus), then that protein would need to go to the nucleus in order to work. However, if a mutation prevents that protein from going to the nucleus, then the protein could no longer carry out its normal function. But how do you know if two structures in a cell are close to each other? And can you tell if their positioning has shifted slightly in a disease state? In order to answer these types of questions, we developed a tool that allows us to investigate how close two structures inside of a cell are to each other. This tool is freely available and can be used by biologists to compare the distribution of any number of structures. We compared it to a leading commercial software product that performs the same function and found that our tool gives similar results. We hope that other researchers find this software pipeline useful for their research.

“Location, location, location. It's critical for real estate and for biological systems.”

**What are the potential implications of these results for your field of research?**

We hope that this tool helps researchers who can't afford to pay licensing fees for commercial software to analyze their data in a unique way. In addition, our software pipeline is shared using tools that are common in data science, such as the Python language, SQL databases, and Jupyter notebooks. By sharing this pipeline, we hope that early career researchers have an easy point-of-entry to learn new skills that could lead to a fulfilling career in data science.

**What has surprised you the most while conducting your research?**

The biggest surprise for me was how much fun it is to code and analyze data! I learned a ton of new skills while conducting this research and it opened a new career path for me (described below).

**Figure:**
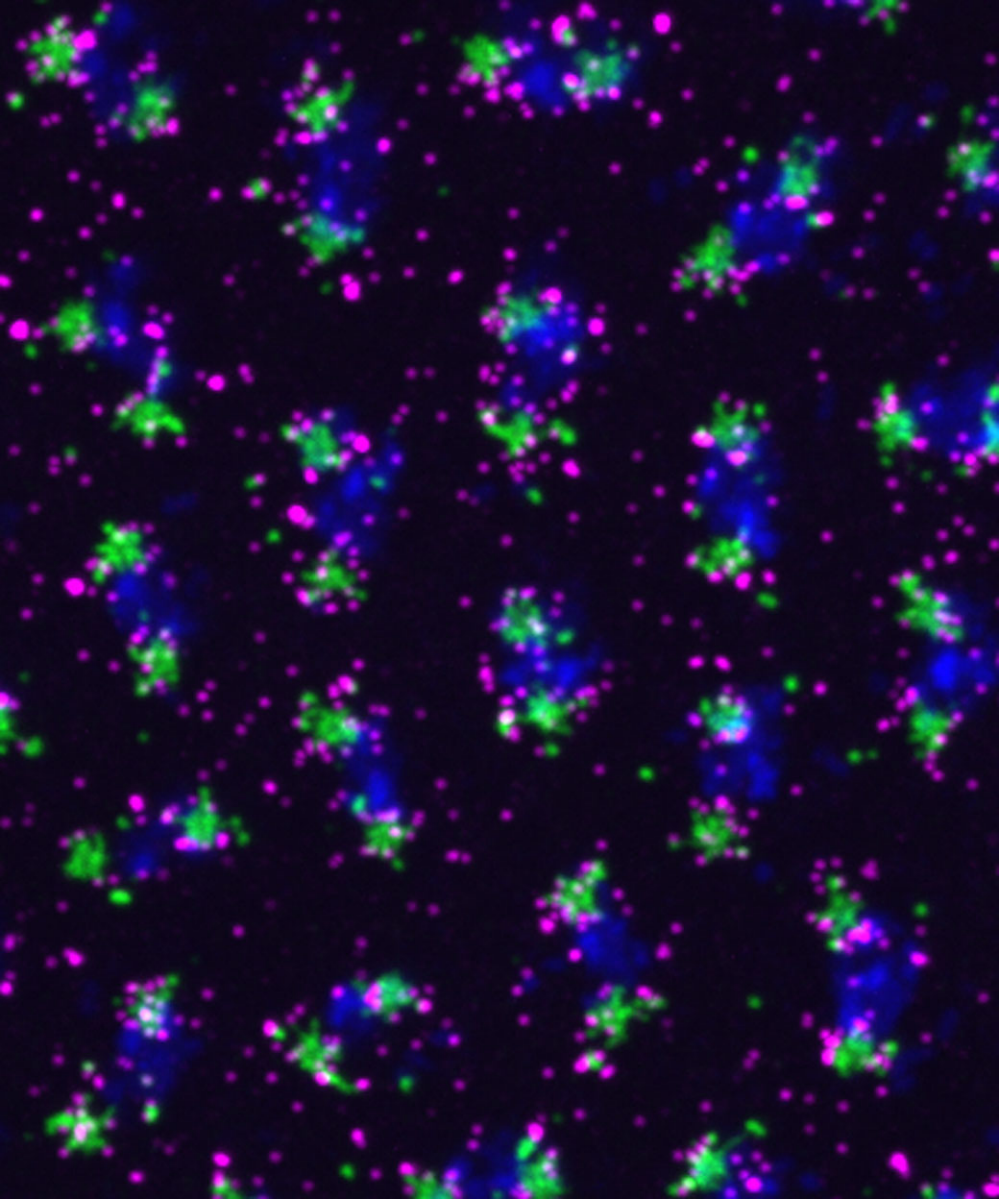
**Research image.**

**What, in your opinion, are some of the greatest achievements in your field and how has this influenced your research?**

I am admittedly biased, but I think that open-source image analysis tools like ImageJ and CellProfiler have hugely revolutionized how biologists can turn images into numbers to extract more knowledge. By making these projects open source, users can contribute new code and further develop these tools for the entire community. Further, both projects are designed to be user friendly with multiple tutorials, which democratizes access to powerful image analysis tools.

**What changes do you think could improve the professional lives of early-career scientists?**

As the founder and moderator of the Future PI Slack group (an online peer mentoring group for postdocs interested in academia), I have a lot of thoughts about this topic. My main focus at the moment is the faculty application process. Candidates spend a lot of time re-writing their job package to fit the different application requirements for each department. For example, one department may ask for a research statement of two pages in length and five letters of recommendation, while another asks for a five-page research statement and three letters of recommendation. While this may sound trivial, applicants commonly apply to more than 50 jobs! I think that if universities and colleges could agree to a standardized Faculty App format, it could be a huge help to candidates on the job market. Instead of thinking about adopting their materials to different formats, they could focus on the more interesting question of tailoring their materials to each job. In addition, I believe that there are amazing candidates who could excel in jobs that they can't apply for due to time constraints (such as being a parent or caring for an elder).

**What's next for you?**

I recently joined the Imaging Platform at the Broad Institute, which is led by Anne Carpenter as a postdoctoral fellow in bioimage analysis. This group created and maintains CellProfiler, an open source tool for image analysis. My role on the assay development team is to collaborate with groups in both industry and academia from around the country to develop methods to analyze imaging data using advanced techniques such as machine learning. I also will work as part of the Center for Open Bioimage Analysis to teach biologists and students how to analyze their imaging data.
